# LuxR-Type SCO6993 Negatively Regulates Antibiotic Production at the Transcriptional Stage by Binding to Promoters of Pathway-Specific Regulatory Genes in *Streptomyces coelicolor*

**DOI:** 10.4014/jmb.2205.07050

**Published:** 2022-09-05

**Authors:** Maral Tsevelkhoroloo, Li Xiaoqiang, Xue-Mei Jin, Jung-Ho Shin, Chang-Ro Lee, Yup Kang, Soon-Kwang Hong

**Affiliations:** 1 Department of Bioscience and Bioinformatics, Myongji University, Yongin 17058, Republic of Korea; 2 GeneNet Pharmaceuticals Co. Ltd., Tianjin 300410, P.R. China; 3 Characteristic Industry Development Center of Yanbian, Jilin Province 133000, P.R. China; 4 R&D, Health & Bioscience, DuPont-IFF, Wilmington 19898, DE, USA; 5 Institute for Medical Sciences, Ajou University School of Medicine, Suwon 16499, Republic of Korea

**Keywords:** *Streptomyces coelicolor*, SCO6993, LuxR family, actinorhodin, undecylprodigiosin

## Abstract

SCO6993 (606 amino acids) in *Streptomyces coelicolor* belongs to the large ATP-binding regulators of the LuxR family regulators having one DNA-binding motif. Our previous findings predicted that *SCO6993* may suppress the production of pigmented antibiotics, actinorhodin, and undecylprodigiosin, in *S. coelicolor*, resulting in the characterization of its properties at the molecular level. *SCO6993*-disruptant, *S. coelicolor* Δ*SCO6993* produced excess pigments in R2YE plates as early as the third day of culture and showed 9.0-fold and 1.8-fold increased production of actinorhodin and undecylprodigiosin in R2YE broth, respectively, compared with that by the wild strain and *S. coelicolor* Δ*SCO6993*/*SCO6993*^+^. Real-time polymerase chain reaction analysis showed that the transcription of *act*A and *act*II-ORF4 in the actinorhodin biosynthetic gene cluster and that of *red*D and *red*Q in the undecylprodigiosin biosynthetic gene cluster were significantly increased by *SCO6993*-disruptant. Electrophoretic mobility shift assay and DNase footprinting analysis confirmed that SCO6993 protein could bind only to the promoters of pathway-specific transcriptional activator genes, *actII*-ORF4 and *red*D, and a specific palindromic sequence is essential for SCO6993 binding. Moreover, SCO6993 bound to two palindromic sequences on its promoter region. These results indicate that *SCO6993* suppresses the expression of other biosynthetic genes in the cluster by repressing the transcription of *actII*-ORF4 and *red*D and consequently negatively regulating antibiotic production.

## Introduction

Streptomycetes are a group of gram-positive, filamentous soil bacteria that exhibit unique morphological differentiation, developing aerial mycelia and spores. During this morphological differentiation, a secondary metabolism begins which is called physiological differentiation. Numerous scientific findings have revealed that the morphological and physiological differentiations in streptomycetes are closely related and controlled by many regulatory factors [1]. The genes responsible for secondary metabolism and morphological differentiation are clustered in the chromosomal DNA and their expressions are controlled by pathway-specific regulatory gene(s) in the cluster and by global regulatory genes.

*Streptomyces coelicolor* produces multiple genetically and structurally distinct antibiotic substances, and the production of pigmented antibiotics, blue-colored actinorhodin and red-colored undecylprodigiosin, is regulated by the pathway-specific transcriptional regulatory genes *act*II-ORF4 [2] and *red*D [3] in the biosynthetic gene cluster. ActII-ORF4 and RedD proteins are the transcriptional activators that bind to the operator regions of the biosynthetic genes and positively regulate the transcription of each gene in the clusters.

Due to easy discrimination of pigmentations in plate culture, several classes of blocked mutants, such as *abs* [4], *afs* [5], and *bld* [6], which block more than one antibiotic, were isolated to identify the global regulatory genes. For example, *afs* (**A**-factor synthesis deficient) mutation results in failure to produce A-factor (γ-butyrolactone), a microbial hormone, and at least four antibiotics, including the two pigmented antibiotics, methylenomycin and Ca^2+^-dependent antibiotic, in *S. coelicolor* A3(2) [7]. The multiple serine/threonine kinases, including AfsK, AfsL, and PkaG phosphorylate AfsR (a central protein that restores afs mutation), and the phosphorylated AfsR bind to the promoter of *afs*S for transcriptional activation. The resulting AfsS protein induces the transcription of pathway-specific transcriptional activators, such as *act*II-ORF4 and *red*D, for actinorhodin and undecylprodigiosin production, respectively [5, 8].

However, the regulation of secondary metabolism appears much more complex than speculated. For example, the transcription of *actII*-ORF4 and *red*D is controlled by other transcriptional activators, AtrA [3] and RedZ [9], respectively. Moreover, *abs*A1/A2 complementing *abs*A (antibiotic synthesis deficient) mutation that fails to produce four distinct antibiotics encodes the bacterial two-component regulatory proteins, the sensor histidine kinase (AbsA1) and response regulator aspartic acid kinase (AbsA2) [4]. The AbsA2 acts as a negative regulator of antibiotic production in *S. coelicolor* A3(2) by binding to the promoter regions of *act*II-ORF4 and *redZ* [10]. Furthermore, tens of activator/repressor proteins have been reported in relation to the transcriptional regulation of *act*II-ORF4 and *red*D in *S. coelicolor* A3(2), indicating the complexity of the regulatory network for secondary metabolism in *Streptomyces* [11].

Recently, we reported that the introduction of *SCO6992* gene encoding a novel β-glucuronidase dramatically increased the biosynthesis of undecylprodigiosin and actinorhodin in *abs*R^-^ mutant of *S. coelicolor* J1501. This antibiotic biosynthesis-promoting effect of *SCO6992* was abolished by the presence of *SCO6993*, which acts in opposition to *SCO6992* [12]. Based on this, *SCO6993* is expected to be a regulatory gene that suppresses antibiotic biosynthesis. Therefore, we report here the regulatory role of the LuxR-type SCO6993 protein in the antibiotic biosynthesis of *S. coelicolor* J1501.

## Material and Methods

### Bacterial Strains and Plasmids

*S. coelicolor* J1501 (*hisA1* *uraA1* *strA1* SCP1^-^ SCP2^-^ Pgl^-^), a mutant strain of *S. coelicolor* A3(2), was kindly provided by Dr. Wendy Champness of Michigan State University [13]. pET28a(+) was used for the expression of *SCO6993* in *E. coli* BL21(DE3)pLysS (Stratagene, USA). *E. coli* ET12567 with the pUZ8002 plasmid was used for conjugal transfer [14]. *E. coli* BW25113 strain with the λred recombination plasmid pIJ790 and cosmid SC8F11 was obtained from the John Inns Institute (UK) and used for genetic recombination [15].

### Media and Culture Conditions

*E. coli* was maintained on M9 minimal agar and cultured in LB medium [16] at 37°C with agitation. *Streptomyces* strains were routinely maintained on minimal medium or R2YE medium [17] at 30°C. For cultivation of *S. coelicolor* J1501, the medium was supplemented with histidine and uracil at final concentrations of 50 μg/ml and 7.5 μg/ml, respectively [12]. Thiostrepton (25 μg/ml), apramycin (50 μg/ml), and kanamycin (50 μg/ml) were added to the media, when necessary.

### Enzymes and Chemicals

DNA-modifying enzymes were purchased from DyneBio Inc. (Korea), and other non-specified fine chemicals were purchased from Sigma-Aldrich Corporation (USA). All primer pairs used in this study were synthesized by Xenotech (Korea) and are listed in Table S1 with their respective uses.

### DNA Manipulations and Transformation

DNA manipulation and transformation were performed by following the methods described by Sambrook and Russel [16] for *E. coli* and Kieser *et al*. [17] for *Streptomyces*. All experimental kits and enzymes were used according to the manufacturer’s recommendations.

### Quantitation of Antibiotics

The production of pigmented antibiotics, actinorhodin and undecylprodigiosin, on plates, was observed by naked eye and identified based on the color change during the cultivation of *Streptomyces* strains on the R2YE medium. For actinorhodin and undecylprodigiosin quantitation in broth culture, an exponential culture (5 ml) of *S. coelicolor* was transferred to 100 ml of R2YE broth in a 500 ml baffled flask and incubated at 30°C on a reciprocal shaker. Portions (5.0 ml) of the culture broth were taken out at intervals and the concentrations of actinorhodin and undecylprodigiosin were measured at 633 nm (A_633_) and 530 nm (A_530_), respectively, as previously described [12].

### Construction of Δ*SCO6993* Mutant

The *SCO6993*-disrupted mutant was constructed by replacing all of the *SCO6993* coding sequence with apramycin resistance cassette [*aac(3)IV*] using the redirect polymerase chain reaction (PCR) targeting method [18]. The upstream primer for Δ*SCO6993* contains the *SCO6993* sequence up to the start codon (in bold) and is linked to the *aac(3)IV* sequence (underlined); 5′-GGG GGG TAC TCA AGT CAC CCT GTA TCA GGG ATG AGT **ATG** ATT CCG GGG ATC CGT CGA CC-3′). The downstream reverse primer contains the Δ*SCO6993* stop codon (in bold) and is linked to the *aac(3)IV* sequence (underlined); 5′-GGG CGG CCT GAG CCC GCC CGG TGC CCG GCC GCC GGG **TCA** TGT AGG CTG GAG CTG CTT C-3′. The resulting PCR product was introduced by electroporation into the *E. coli* BW25113 strain that harbors the λ*red* recombination plasmid pIJ790 and cosmid SC8F11 (a gift from K. Chater at John Innes Centre) carrying *SCO6993* [15]. The gene structure of the resulting cosmids (SC8F11 Δ*SCO6993::apr*) recovered from the selected transformants was verified, and the cosmids were introduced into the *E. coli* ET12567 carrying pUZ8002 and then transferred into *S. coelicolor* J1501 by conjugation [18]. The apramycin-resistant and kanamycin-sensitive exconjugants were selected, and the expected gene structure in Δ*SCO6993::apr* mutant was confirmed by Southern hybridization and reverse transcription (RT)-PCR. For complementing *SCO6993* disruption, the recombinant plasmid pWHM3-O2 containing the entire *SCO6993* with its promoter region as described previously [12] was transformed into Δ*SCO6993::apr* mutant, yielding *S. coelicolor* Δ*SCO6993*/*SCO6993*^+^.

### Southern Hybridization

To confirm the Δ*SCO6993::apr* mutant, chromosomal DNAs were isolated and digested using BamHI restriction enzyme. The fully digested genomic DNAs were applied to 0.8% TBE-agarose gel. Resolved DNA fragments were depurinated in 0.25 M HCl for 20 min when required, washed with distilled water briefly, denatured in 0.4 M NaOH for 10 min, and transferred to Hybond-N^+^ membrane (Amersham Life Science, UK) in 0.4 M NaOH. The *aac(3)IV* probe DNA was amplified with DIG-labeled nucleotides and hybridized overnight. The signal was detected before image scanning following the manufacturer’s instructions.

### Preparation of Total RNA and cDNA

The fresh spores of *S. coelicolor* strains were inoculated into 25 ml R2YE broth in 250 ml Erlenmeyer flasks or R2YE plates and incubated at 28°C. The cultured cells were quickly harvested, flash frozen in liquid nitrogen, and then ground into powder. Total RNA was purified using an RNeasy Mini Kit in combination with RNAprotect Bacteria Reagent (Qiagen, Netherlands). The DNA in the RNA sample was removed through on-column DNase digestion using an RNase-Free DNase Set (Qiagen). Then, cDNA was synthesized from the total RNA sample using the SuperScript III First-Strand Synthesis System for RT-PCR (Invitrogen, USA). Annealing reaction was performed at 25°C for 10 min, and then RT was performed at 50°C for 50 min. Reverse transcriptase was inactivated by heat treatment (85°C, 5 min). After cooling at 4°C, the remaining RNA was removed by RNase H treatment (37°C, 20 min). The synthesized cDNA was stored at -80°C.

### Reverse Transcription-Polymerase Chain Reaction (RT-PCR)

To check the transcription level of *S. coelicolor* Δ*SCO6993*, RT-PCR was performed using the synthesized cDNA, primers (Table S1), and rTaq Plus 5x PCR Master Mix (ELPIS Biotech, Korea). After denaturation at 95°C for 3 min, the PCR reaction (denaturation at 95°C for 10 s; annealing at 68°C for 10 s; and extension at 72°C for 10 s) was repeated 28 times. The housekeeping *hrdB* gene encoding RNA polymerase sigma factor was used as control.

### Real-Time PCR Analysis

To check the transcription level of the genes related to actinorhodin and undecylprodigiosin biosynthesis, real-time PCR analysis was performed using the synthesized cDNA and 2x Real-Time PCR Premix with Evagreen (SolGent, Korea). *act*A (encoding an efflux pump) and *act*II-ORF4 (encoding a transcriptional activator) were chosen among the actinorhodin biosynthetic genes, and *red*D (encoding a transcriptional activator) and *red*Q (encoding an acyl carrier protein) were chosen from the undecylprodigiosin biosynthetic gene clusters. The reaction was performed at 95°C for 10 min followed by 40 cycles of 95°C for 20 s, 58°C for 40 s, and 72°C for 20 s in a 20 μl volume mix containing 1 μl of 20x EvaGreen, 0.25 μM primers (Table S1), and 40 ng cDNA. Thermocycling and fluorescence detection were performed in triplicates for each cDNA sample using the Stratagene Mx3000p Real-Time PCR System (Stratagene). Following amplification, the experiment was converted to a comparative quantification (calibrator) experiment type and analysis was performed using the Mx3000P software v2.02 (Stratagene). The housekeeping *hrdB* gene was used as a reference.

### Heterologous Expression and Purification of SCO6993

The *SCO6993* gene was amplified by PCR from the *S. coelicolor* J1501 genomic DNA using primer sets (Table S1). The NdeI-XhoI-digested PCR product was subcloned into pET28a(+) yielding pET28a-SCO6993. *E. coli* BL21 (DE3)/pET28a-SCO6993 was cultured in 200 ml of LB broth to an optical density at 600 nm (OD600) of 0.5. The overexpression of *SCO6993* was induced by adding IPTG (0.5 mM), and there was further cultivation at 16°C for 12 h. From the cells, the recombinant SCO6993 (rSCO6993) protein was purified using His60 Ni Superflow Resin & Gravity Columns (Takara Bio Company, Japan). Protein concentration was measured using a Bradford Protein Microassay Kit (Bio-Rad, USA) with bovine serum albumin as the standard protein.

The molecular mass of the purified rSCO6993 was determined by gel filtration chromatography. The ÄKTA-FPLC System (GE Healthcare Life Sciences, USA) equipped with a Superose 12 10/300 GL column was used at 25°C. The mobile phase (20 mM Tris-HCl (pH 7.9) + 100 mM NaCl) was applied at a flow rate of 0.7 ml/min and protein concentration was monitored at 280 nm.

### Electrophoretic Mobility Shift Assay (EMSA)

The binding ability of rSCO6993 to the promoter regions of *SCO6993*, *act*A, *actII*-ORF4, *red*Q, and *red*D was evaluated using the EMSA. For the *SCO6993*, two kinds of DNA probes, *SCO6993*-P1 (206 bp) and *SCO6993*-P2 (192 bp), were amplified from the *S. coelicolor* J1501 genomic DNA by PCR using primer sets (Table S1). Other probes for *act*A, *act*II-ORF4, *red*Q, and *red*D were also prepared in the same way. Binding reactions (20 μl) were performed in binding buffer (20 mM Tris-HCl (pH 8.0), 1 mM MgCl_2_, 0.1 mM dithiothreitol, 12.5% glycerol, 50 mM NaCl, and 0.4 mM EDTA) with a 0.3 nM DNA probe at 28°C for 30 min. The sonicated salmon sperm DNA (100 mg/ml) was used as a competitor and the binding of the SCO6993 to the DNA fragments was analyzed using 1.5% TBE agarose gel.

### Mapping of the SCO6993-Binding Sites

DNase 1 footprinting analysis was performed with capillary DNA sequencer using a fluorescent-labeled PCR fragment as previously described [19]. Primers for analysis of each promoter region were used by labeling the same primers as those used in the EMSA experiment with 5' 6-FAM (6-carboxyfluorescein) (Table S1). The labeled DNA probes (540 ng) were incubated with the SCO6993 protein (25 μg) at 28°C for 20 min in the binding buffer (20 mM Tris-HCl (pH 8.0), 1 mM MgCl2, 0.1 mM DTT, 12.5% glycerol, 50 mM NaCl, and 0.4 mM EDTA). The digestion of the DNA probe was performed at 37°C using one unit of DNase 1 (Promega, USA) per 50 μl reaction solution. After 5 min, a stop buffer (130 mM NaCl, 20 mM EDTA, and 0.6% SDS) was added, and the digested DNA probe was purified using a PCR purification kit (Qiagen). The fluorescence patterns of the sample were analyzed using a 3730 Automated DNA Sequencer (Applied Biosystems, USA), and electropherograms were used for determining the protected patterns of each promoter after digestion with DNase 1 following incubation with SCO6993 protein.

## Results

### SCO6993 Belongs to the Large ATP-Binding Regulators of the LuxR (LAL) Family

**Fig. 1:**
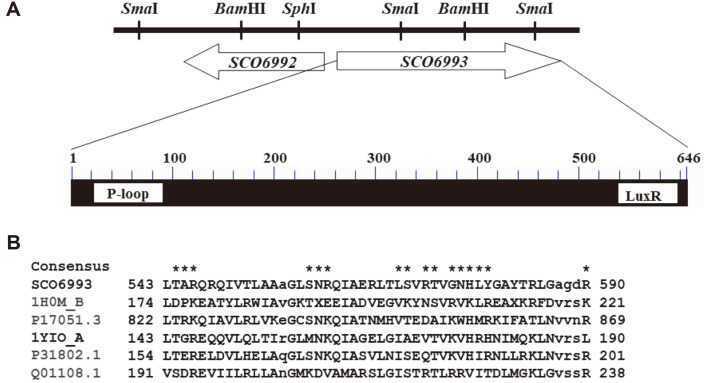
Characteristics of *SCO6993* gene in *S. coelicolor* J1501. (**A**) Chromosomal gene organization (upper) and distribution of conserved domains (lower). The arrows indicate individual ORFs, with the stop codon marked by the arrowhead. The *SCO6992* and *SCO6993* genes are in opposite directions, and the two genes are 78 bp apart from each putative transcription initiation point. SCO6993 (646 amino acids) contains ATP/GTP-binding P-loop domain (Phe-23 ~ Leu-135, Evalue of 2.36e^-03^) and one LuxR-type helix-turn-helix DNA binding domain (Leu-543 ~ Arg-590, E-value of 6.58e^-14^). (**B**) Alignment of amino acids sequence of C-terminal DNA-binding domain of LuxR-like proteins (cd06170). Highly conserved amino acids for DNA binding are indicated by asterisks. 1H0M_B; a transcriptional activator protein TraR from *Agrobacterium tumefaciens*, P17051.3; a transcriptional regulator AlkS from *Pseudomonas oleovorans*,1YIO_A; a regulator StyR from *Pseudomonas fluorescens*, P31802.1; a nitrate/nitrite response regulator protein NarP from *Escherichia coli* K-12, Q01108.1; a bialaphos biosynthetic pathway regulatory protein from *Streptomyces hygroscopicus*.

The SCO6993 protein (606 amino acids, 65.7 kDa, GenBank AAF19104.1) showed significant similarity with numerous proteins annotated as LuxR-type, DNA-binding regulators in the genus *Streptomyces*. BlastP analysis (BLAST: Basic Local Alignment Search Tool (nih.gov)) identified more than 100 proteins showing more than 98%homology to SCO6993 in the amino acid sequence in *Streptomyces*, implying that the SCO6993 homologous gene is widely distributed in genus *Streptomyces*. SCO6993 has one ATP/GTP-binding P-loop motif conserved in ATPase and one helix-turn helix LuxR-type DNA-binding motif in the C-terminal region, which is a characteristic of the LAL family protein (Fig. 1). In this context, SCO6993 was expected to be a DNA-binding regulatory protein.

### *SCO6993* Gene Deletion Results in Increased Production of Pigmented Antibiotics

**Fig. 2:**
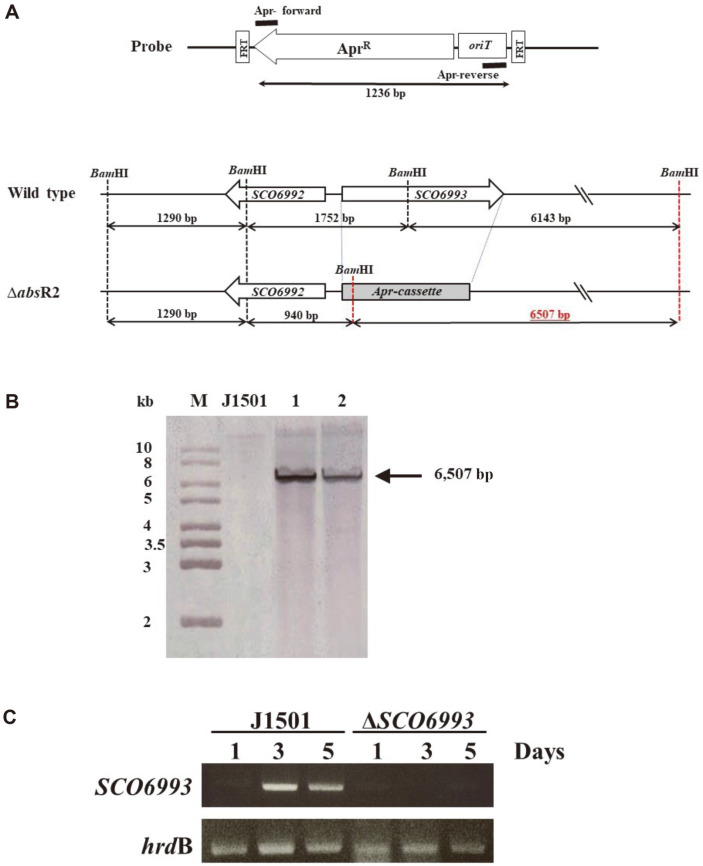
Disruption of the chromosomal *SCO6993* gene in *S. coelicolor* J1501. (**A**) Schematic representation of disruption. The sizes of the expected fragments of the disruptant with probe 1 and probe 2 are indicated. *apr*, apramycin resistance. (**B**) Genomic Southern blotting analysis for confirming Δ*SCO6993* mutant strain. The sizes of the DNA marker bands are shown on the left. The detected signals represent hybridized DNA fragments with DIG-labeled *apr*-cassette probe (1,236 bp). The hybridized signal of the expected size (6,507 bp) was observed in ΔSCO6993 mutant (lane 1 and 2) but not in the wild type. (**C**) Reverse transcription polymerase chain reaction analysis for confirming Δ*SCO6993* mutant strain cultured on R2YE plate. In *S. coelicolor* J1501, a large amount of the *SCO6993* gene transcript was detected on the third day (maximum level) and the fifth day of cultivation, but no transcript was detected in the Δ*SCO6993* mutant strain. The housekeeping *hrdB* gene encoding RNA polymerase sigma factor was used as control.

In a previous study [12], SCO6993 was expected to be a suppressor for pigmented antibiotic production. Thus, *SCO6993*-disrupted strain (Δ*SCO6993*) was constructed using a PCR targeting system (Fig. 2A). For the apramycin-resistant and kanamycin-sensitive exconjugants, Δ*SCO6993::apr* mutant was confirmed using Southern blotting and hybridization with a DIG-labeled apramycin-cassette probe (Fig. 2B). According to the RT-PCR analysis, the transcript of *SCO6993* in the *S. coelicolor* J1501 on R2YE plate was maximally detected on the third day of cultivation, and a significant amount was also detected on the fifth day. However, no transcript of *SCO6993* was detected in the Δ*SCO6993* strain during cultivation (Fig. 2C), indicating that the *SCO6993* gene in the Δ*SCO6993::apr* mutant completely lost its function.

**Fig. 3:**
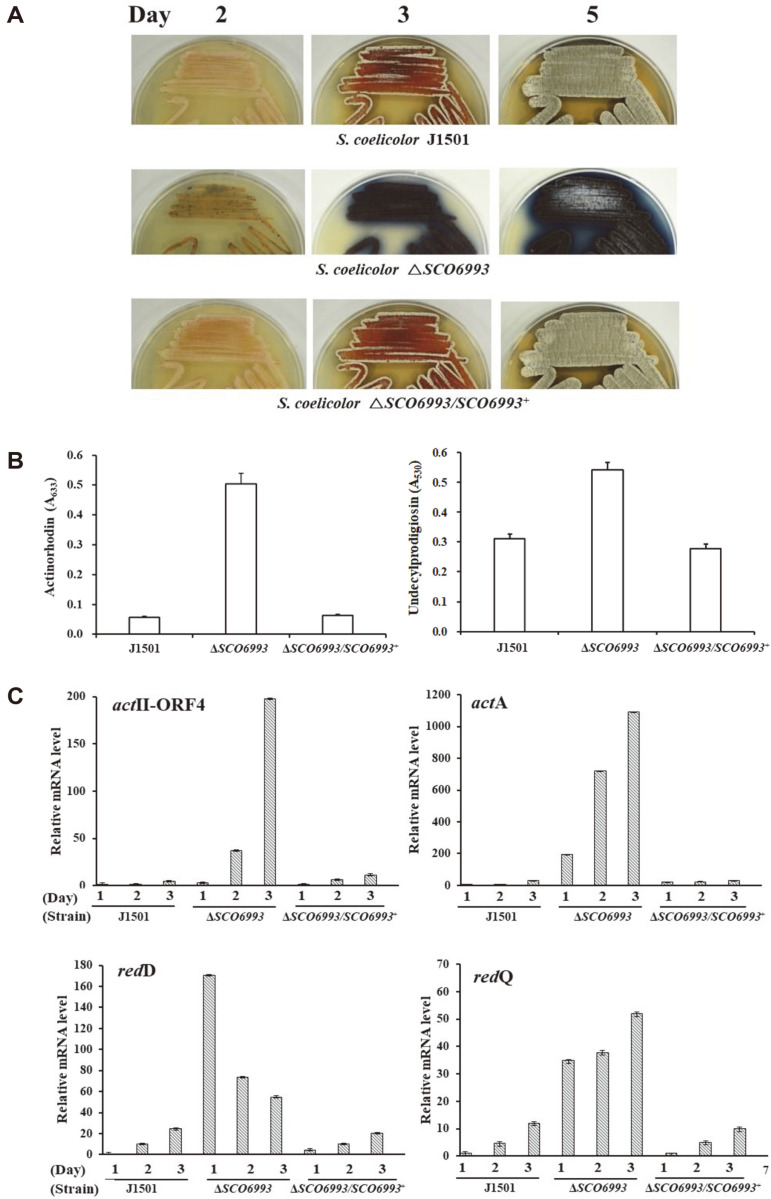
Effect of *SCO6993* disruption on pigmented antibiotic production in *S. coelicolor* J1501. (**A**) Photographs of *S. coelicolor* strains cultured on R2YE agar plate for 5 days. *S. coelicolor* Δ*SCO6993* showed a large amount of pigment production from the third day of culture, but the parent strain J1501 and the mutant complemented with *SCO6993* (*S. coelicolor* Δ*SCO6992/SCO699*^2+^) produced a small amount of pigment even on the fifth day. (**B**) Pigmented antibiotic production in R2YE broth. The culture samples were taken on the seventh day of cultivation and processed. The quantification of actinorhodin (left) and undecylprodigiosin (right) was performed by measuring the absorbance at 633 nm and 530 nm, respectively. (**C**) Transcript analysis by real-time polymerase chain reaction for actinorhodin (upper) and undecylprodigiosin (lower) biosynthetic genes of *S. coelicolor* strains cultured on R2YE agar plate. The culture samples were taken at indicated time intervals and processed as described in the Materials and Methods section. Then, the experiment was converted to a comparative quantification (calibrator) experiment and analyzed with the Mx3000P software v2.02. The housekeeping *hrd*B gene was used as a reference. In (**B**) and (**C**), all the experiments were repeated at least three times, and their average values were calculated. J1501, *S. coelicolor* J1501; Δ*SCO6993*, *S. coelicolor* Δ*SCO6993*; Δ*SCO6993*/*SCO6993*^+^, *S. coelicolor* Δ*SCO6993* complemented with native *SCO6993* gene.

The antibiotic production ability of the Δ*SCO6993* mutant was compared to that of the parent strain by culturing in solid and liquid media. On the R2YE plates, little pigment production was observed in the J1501 parental strain, while the Δ*SCO6993* mutant continuously produced excess pigments from the third day of culture (Fig. 3A).

In the R2YE liquid culture, the Δ*SCO6993* strain began producing large amounts of pigment from the third day of culture. On the seventh day, the Δ*SCO6993* strain showed 9.0-fold and 1.8-fold increased production of actinorhodin and undecylprodigiosin, respectively, compared to that by the *S. coelicolor* J1501 strain (Fig. 3B). The *SCO6993*-complemented *S. coelicolor* Δ*SCO6993*/*SCO6993*^+^ strain had its normal phenotype restored as in J1501 in solid (Fig. 3A) and liquid media (Fig. 3B).

### Transcriptions of Pigmented Antibiotic Biosynthetic Genes Are Enhanced in *SCO6993*-Deleted Strain

To understand the reason for the increased production of pigmented antibiotics in Δ*SCO6993* strain, real-time PCR analysis was performed on the two genes (*act*A and *act*II-ORF4) in the actinorhodin biosynthetic gene cluster and the two genes (*red*D and *red*Q) in the undecylprodigiosin biosynthetic gene cluster. The real-time PCR analysis showed that the transcription of all the tested genes was significantly enhanced in the Δ*SCO6993* strain from the beginning of the culture in R2YE broth, compared with that in J1501 and *S. coelicolor* Δ*SCO6993*/*SCO6993*^+^ (Fig. 3C).

The transcription of *act*II-ORF4 increased in proportion to the cultivation time, but that of *red*D showed a maximum level on the first day of culture. When observing the R2YE plate culture of these strains, the production of red-colored undecylprodigiosin was visually confirmed from the first day of culture, and the production of blue-colored actinorhodin was observed from the third day of culture (Fig. 3A). These results strongly suggest that undecylprodigiosin production begins temporally faster than actinorhodin production.

### SCO6993 Binds to the Promoter Regions of *act*II-ORF4 and *red*D Regulatory Genes

**Fig. 4:**
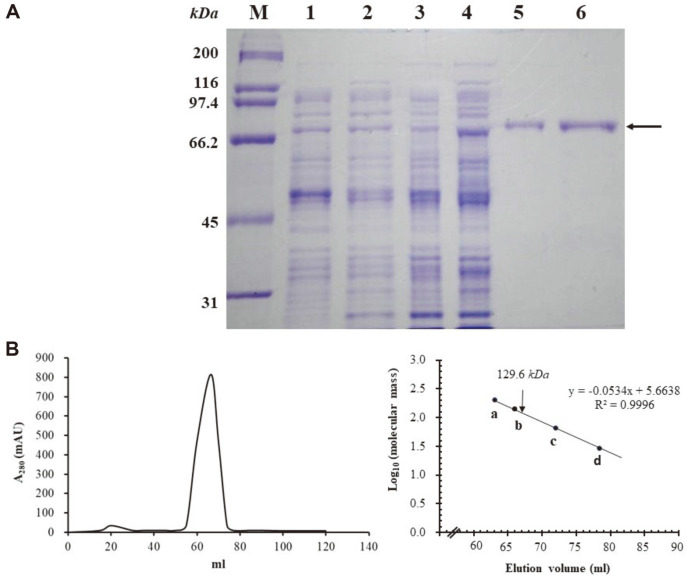
Purification and molecular weight estimation of SCO6993. (**A**) Sodium dodecyl sulfate polyacrylamide gel electropherogram. rSCO6993 (His_6_-SCO6993) was overexpressed in *E. coli* BL21 (DE3)/pET28a-SCO6993 and purified by His60 Ni Superflow Resin chromatography as indicated by an arrow. Lanes: M, molecular weight size markers; 1, *E. coli* BL21 (DE3)/pET28a before induction; 2, *E. coli* BL21 (DE3)/pET28a after induction; 3, *E. coli* BL21 (DE3)/pET28a-SCO6993 before induction; 4, *E. coli* BL21 (DE3)/pET28a-SCO6993 after induction; 5, purified His_6_-SCO6993 protein; and 6, purified His_6_-SCO6993 protein after dialysis. (**B**) Gel-filtration chromatogram. (Left) The purified rSCO6993 protein (2.5 mg/ml) was applied on HiLoad 16/60 superdex 200 prep-grade column and the elution profile was monitored at 280 nm (A_280_). (Right) The molecular mass of rSCO6993 was calculated from the elution profile of the size marker proteins: position a, β-amylase (200 kDa); position b, yeast alcohol dehydrogenase (150 kDa); position c, bovine serum albumin (66 kDa); position d, bovine carbonic anhydrase (29 kDa). Blue dextran 2000 was used to determine the column void volume (V_0_). The position of rSCO6993 elution peak is indicated with an arrow.

To determine the DNA-binding ability of the LuxR-type regulator, the recombinant protein rSCO6993 was overexpressed with 20 additional amino acids including 6xHis-tag, which originated from the vector at the N-terminal. The purified rSCO6993 protein showed a single band by SDS-PAGE analysis, which is consistent with a calculated molecular weight of 66.8 kDa (Fig. 4A). The gel filtration chromatography analysis revealed that the molecular weight of native SCO6993 was 129.6 kDa, indicating that it forms a dimer (Fig. 4B).

**Fig. 5:**
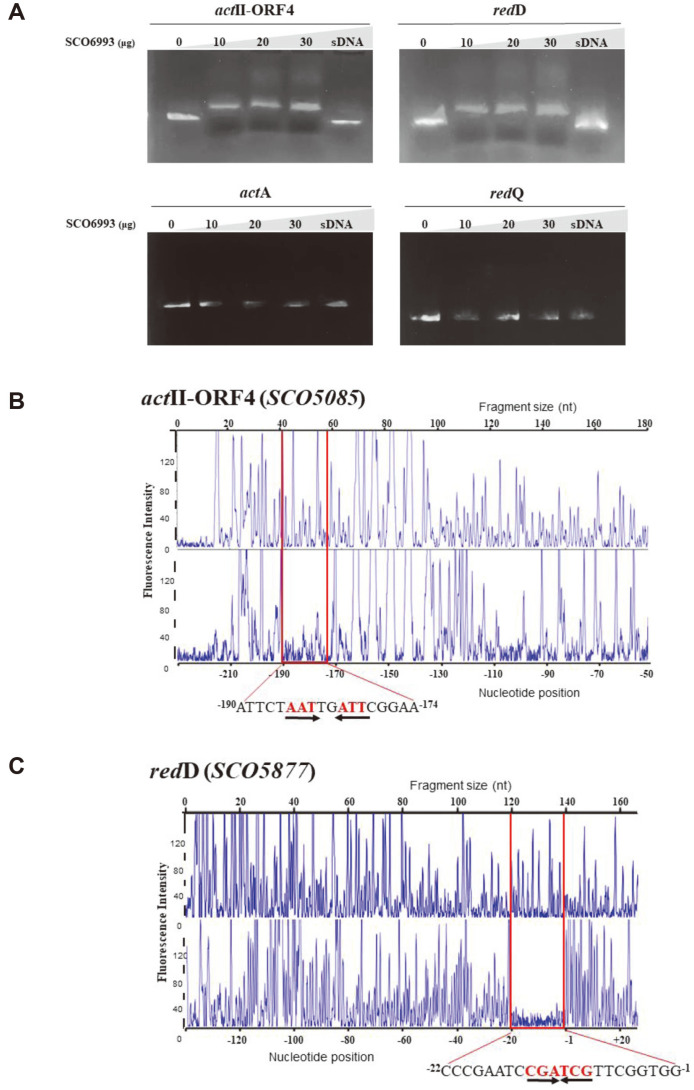
DNA-binding ability of SCO6993 to the promoter regions of actinorhodin (*act*) and undecylprodigiosin biosynthetic genes. (**A**) Electrophoretic mobility shift assays (EMSAs) were performed with purified His_6_-SCO6993 on the promoter regions of the genes involved in actinorhodin (*act*) and undecylprodigiosin (*red*) biosynthesis. The DNA probes used were the upstream regions of *actII*-ORF4 (p*actII*-ORF4), *act*A (p*act*A), *red*D (p*red*D), and *red*Q (predQ). Each 20 μl binding reaction mixture containing the DNA probe (0.3 nM) and the SCO6993 protein at various concentrations (0–30 μg), was subjected to electrophoresis on 1.5% TBE agarose gel. sDNA, salmon sperm DNA. (**B, C**) DNase 1 footprinting assays. Electropherograms showing the protection pattern of each promoter of the fluorescent dye-labeled upstream regions of actIIORF4 (**B**) and *red*D (**C**) after digestion with DNase 1 following incubation in the absence (upper) or presence (lower) of SCO6993 protein. The y-axis represents fluorescence intensity (**i.e.**, fragment abundance), while the x-axis represents elution position, which is proportional to size. At the bottom is a scale that shows nucleotide position relative to each gene. The protected regions are indicated with a red box, and the protected sequences (SCO6993-binding sites) with palindrome sequences are shown below. The numbers refer to the distance from the translation start site of each gene.

Next, a DNA fragment containing the upstream regions of *act*A, *act*II-ORF4, *red*D, and *red*Q served as the binding partner of rSCO6993 in the EMSAs. DNA fragments of 327 bp (p*actII*-ORF4; between positions -207 and+120 with respect to the *actII*-ORF4 translation start site), 383 bp (p*act*A; between positions -273 and +110 with respect to the *act*A translation start site), 400 bp (p*red*D; between positions -350 and +50 with respect to the *red*D translation start site), and 350 bp (p*red*Q; between positions -300 and +27 with respect to the *red*Q translation start site) were used as DNA probes for *act*II-ORF4, *act*A, *red*D, and *red*Q, respectively (Table S1). As a result, the retardation of DNA mobility for the two pathway-specific genes, *act*II-ORF4 and *red*D, was confirmed, but not for biosynthetic genes *act*A and *red*Q (Fig. 5A). The intensity of the retardation signal increased in proportion to the concentration of added SCO6993. The addition of salmon sperm DNA (100 mg/ml) to the reaction inhibited complex formation between SCO6993 and the DNA substrate.

### SCO6993 Binds to Palindromic Sequences in the Promoter Regions of *actII*-ORF4 and *red*D Genes

SCO6993-binding sites within the promoter regions of *actII*-ORF4 and *red*D were validated using DNase 1 footprinting analysis. The electropherograms showed the protected patterns of each promoter after digestion with DNase 1 following incubation with SCO6993 protein. The results showed that the region of *actII*-ORF4 promoter protected by SCO6993 was from -190 to -174 nt (^-190^ATTCT**AAT**TG**ATT**CGGAA^-174^, palindromic sequence is underlined) (Fig. 5B), whereas the region of *red*D promoter protected by SCO6993 was -22 to -1 nt (^-22^CCCGAA TC**CGATCG**TTCGGTGG^-1^, palindromic sequence is underlined) (Fig. 5C). The binding motif of most LuxR family transcriptional regulators is palindromic, but highly degenerate due to sequence variations in each promoter [20]. Similarly, the binding sequences in the promoter regions of *actII*-ORF4 and *red*D comprise palindromes; however, no homology was found between the two.

In summary, SCO6993 appears to suppress the transcription of the pathway-specific regulatory genes *actII*-ORF4 and *red*D by binding directly to their promoter regions. Accordingly, the transcription of the antibiotic biosynthesis genes *act*A and *red*Q will be regulated in succession by the pathway-specific transcriptional activators ActII-ORF4 and RedD, respectively.

### SCO6993 Binds to Its Own Promoter Region

**Fig. 6:**
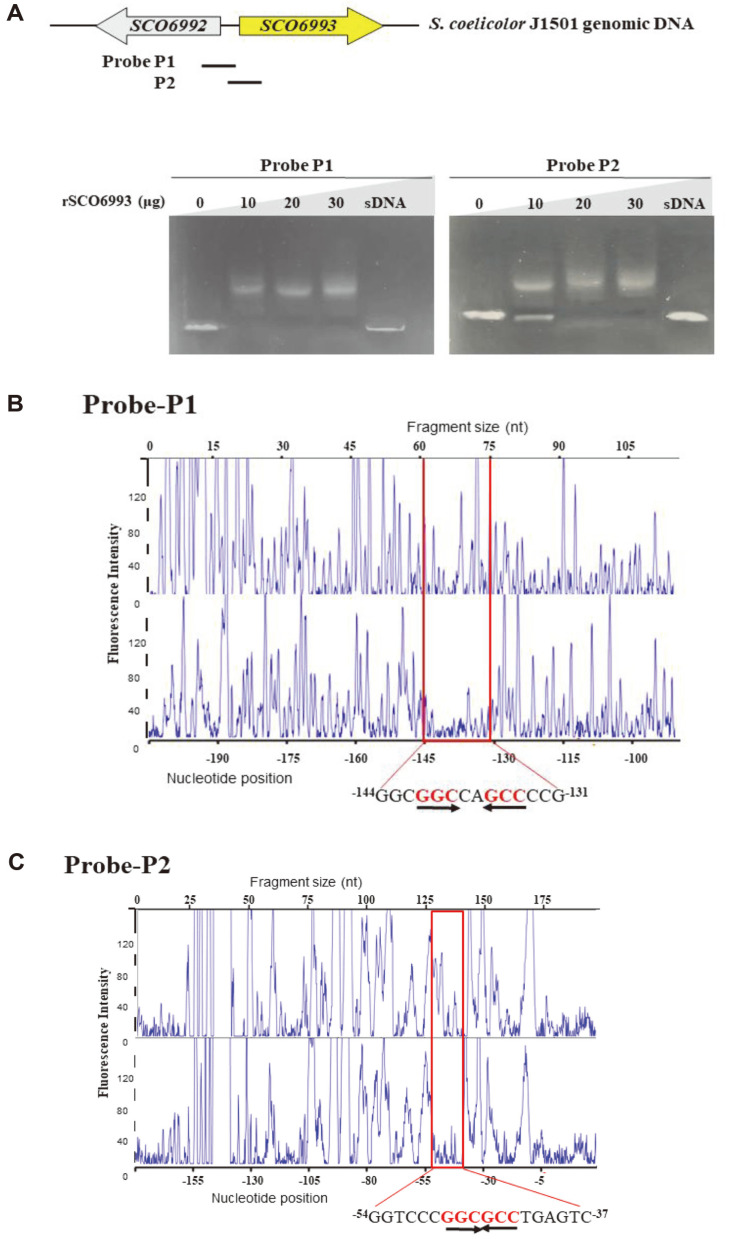
DNA-binding ability of SCO6993 to its promoter region. (**A**) Electrophoretic mobility shift assays (EMSAs) were performed with purified His_6_-SCO6993 on the promoter regions of *SCO6993*. The DNA probes were SCO6993-P1 (-240 ~ -34) and SCO6993-P2 (-55 ~ +114). Each 20 μl binding reaction mixture containing the DNA probe (0.3 nM) and the SCO6993 protein at various concentrations (0–30 μg) was subjected to electrophoresis on 1.5% TBE agarose gel. sDNA, salmon sperm DNA. (**B, C**) DNase 1 footprinting assays using Probe-P1 (**B**) and Probe-P2 (**C**). Electropherograms showing the protection pattern of each promoter of fluorescent dye-labeled upstream region of *SCO6993* after digestion with DNase 1 following incubation in the absence (upper) or presence (lower) of SCO6993 protein. The *y*-axis represents fluorescence intensity (*i.e.*, fragment abundance), while the x-axis represents elution position, which is proportional to size. At the bottom is a scale that shows the nucleotide position relative to each gene. Two protected regions are indicated with a red box, and the protected sequences (SCO6993-binding sites) with palindrome sequences are shown below. The numbers refer to the distance from the translation start site of each gene.

Many transcriptional regulatory proteins can bind to their respective promoter for self-regulation and thus EMSA was performed on the promoter region of *SCO6993* gene. The shifted bands by retarded mobility were detected in all the reactions using probes SCO6993-P1 (207 bp; between positions -240 and -34 with respect to the *SCO6993* translation start site) and SCO6993-P2 (192 bp; between positions -78 and +114 with respect to the *SCO6993* translation start site), and the amount of shifted DNA was proportional to that of protein added (Fig. 6A). The addition of excess salmon sperm DNA abolished the band shift of DNA. Consistent with the EMSA results, DNase 1 footprinting analysis confirmed the two SCO6993-binding sites. The regions of *SCO6993* promoter protected by SCO6993 were from -144 to -131 nt (^-144^GGC**GGC**CA**GCC**CCG^-131^, palindromic sequence is underlined) and from -54 to -37 nt (^-54^GGTCCC**GGCGCC**TGAGTC^-37^, palindromic sequence is underlined) as we expected (Figs. 6B and 6C). These results strongly suggest that SCO6993 can self-regulate the expression of its encoding genes by binding to its promoter region.

## Discussion

In this study, we demonstrated that SCO6993, a LAL-type global regulator, suppressed actinorhodin and undecylprodigiosin production by binding to the promoter regions of the pathway-specific regulatory genes *actII*-ORF4 and *red*D, respectively, in *S. coelicolor* J1501 and repressing their transcription.

LuxR family proteins were identified as transcriptional regulators for quorum sensing-related genes [21, 22] in *Vibrio fischeri*. They have a DNA-binding domain (helix-turn-helix motif) at the C-terminal module for binding specific nucleotide sequences near target promoters and the N-terminal domain for binding effector molecules, thereby regulating the transcription of target genes [23, 24]. In the quorum-sensing regulation, the *N*-acyl derivatives of homoserine lactone (Acyl HSL) binds to the N-terminal domain and triggers LuxR dimerization. Because A-factor (homoserine lactone) was reported as a microbial hormone to control physiological and morphological differentiations in *Streptomyces* including *S. coelicolor* [7], it may be listed as one of the plausible effector candidates for SCO6993. Notably, the binding ability of rSCO6993 to its promoter region was not affected by D-glucose, D-galactose, and D-glucuronic acid (Fig. S1).

The LAL family regulator MalT was first reported in the maltose regulon in *E. coli* [25]. LAL regulators are characterized by a big molecular size due to the presence of an N-terminal ATP/GTP-binding domain (Walker A motif) [26] and a C-terminal LuxR-type DNA-binding domain, which are well conserved in SCO6993. MalT positively regulates the transcription of the maltose regulon by its specific binding to the short DNA motif, called the MalT box (5′-GGA[TG]GA-3′), in the promoter region of its target genes. [27]. By contrast, numerous LuxR family regulators recognize and bind to specific target binding sites with inverted repeats. For instance, LuxR dimer involved in quorum sensing binds to a 20-bp inverted repeat [22], and GerE dimers controlling respiratory-related operons in *E. coli* bind to two 12-bp inverted repeats having a central four-base overlap [28]. Our results indicate that SCO6993 can recognize specific nucleotide sequences containing short, inverted repeats, but the consensus binding site could not be predicted.

Multiple LAL family regulators have been reported to be present in the biosynthetic gene clusters of the secondary metabolites in *Streptomyces*. PikD [29], RapH [30], NysRI [31], TmcN [32], SanG [33], TrdH [34], and AmphRI [35] were identified as LAL family pathway-specific regulators in pikromycin, rapamycin, nystatin, tautomycetin, nikkomycin, tirandamycin, and amphotericin biosynthetic pathways, respectively. Furthermore, LAL regulators play a global regulatory role at higher levels of the regulatory cascade. For example, LAL regulator PimR activates PimM expression in *Streptomyces natalensis* and LuxR-type pathway-specific regulator PimM directly stimulates the transcription of pimaricin biosynthetic genes [36], which is very similar to the relationship between SCO6993 and pathway-specific regulatory genes *actII*-ORF4 and *red*D.

Most LAL regulators function as positive or negative regulators, but some of them act simultaneously as positive and negative regulators depending on the regulatory pathway. In *S. coelicolor*, the LAL regulators SCO0877 and SCO7173 function as repressors of the expression of the two-component PhoRP system in phosphate starvation response and as activators of actinorhodin biosynthesis [37]. The pathway-specific regulator AveR was also reported to be a positive regulator of avermectin production but a negative regulator of oligomycin biosynthesis in *Streptomyces avermitilis* [38]. Interestingly, we found that the ability of aerial mycelium formation was completely abolished by deleting the *SCO6993* in *S. coelicolor* (Figs. S2 and S3), indicating that SCO6993 regulates morphological differentiation positively like the *bld* genes [6, 39]. Thus, SCO6993 can be defined as a global regulatory protein acting negatively or positively in controlling the physiological and morphological differentiations of *S. coelicolor* J1501.
